# Vision and implementation for the first U.S. carbon ion therapy center part I: Clinical rationale and the need for CIRT in the United States

**DOI:** 10.1002/acm2.70876

**Published:** 2026-10-28

**Authors:** Xiaoying Liang, Chris J Beltran

**Affiliations:** ^1^ Department of Radiation Oncology Mayo Clinic Jacksonville Florida USA

**Keywords:** carbon ion radiotherapy, clinical indications, united states

## Abstract

Carbon ion radiotherapy (CIRT) has evolved into an established clinical modality over the past three decades. Owing to its unique physical and biological characteristics, CIRT has demonstrated favorable outcomes for selected malignancies characterized by radioresistance, proximity to critical organs, locally advanced disease requiring safe dose escalation, and recurrent tumors requiring re‐irradiation. This review summarizes the physical and radiobiological rationale for CIRT, the evolution of international clinical practice and the clinical relative biological effectiveness models and dose prescription systems currently used in Asia and Europe. We discuss the differences between the modified microdosimetric kinetic model and Local Effect Model frameworks and their implications for interpreting and applying international clinical experience. We also describe the biological dose calculation framework adopted by Mayo Clinic Florida (MCF) to integrate established international CIRT experience into the first U.S. program. Published estimates of CIRT eligibility and need in the United States are reviewed to provide context for the anticipated U.S. patient population. This population includes many clinically complex disease sites characterized by proximity to critical organs, respiratory motion, anatomical variation, and the potential need for adaptive treatment. These clinical characteristics define important requirements for the first U.S. CIRT program and provide the foundation for the design philosophy and technical implementation described in the companion Part II article.

## 1 INTRODUCTION

Radiotherapy is a cornerstone of cancer treatment, with the primary objective of maximizing tumor control while minimizing toxicity to surrounding normal tissues. Despite major advances in radiotherapy, treatment outcomes remain suboptimal for several malignancies, particularly those that are intrinsically radioresistant, hypoxic, located adjacent to critical normal structures, or recurrent after prior irradiation. Carbon ion radiotherapy (CIRT) has emerged as a promising treatment modality for these challenging clinical cases because it combines enhanced biological effectiveness with favorable dose conformity enabled by the pronounced Bragg peak and reduced lateral scattering of carbon ion beams.

Over the past three decades, CIRT has evolved into an established clinical modality. Seventeen treatment centers are now in clinical operation worldwide, and clinical experience has expanded substantially, with growing evidence demonstrating favorable outcomes across a range of difficult‐to‐treat malignancies.^1^, ^2^, ^3^, ^4^, ^5^, ^6^, ^7^, ^8^, ^9^, ^10^, ^11^, ^12^, ^13^, ^14^, ^15^, ^16^At the same time, advances in technology, treatment planning, image guidance, motion management, and radiobiological modeling have substantially enhanced the precision and clinical capabilities of CIRT.^17^, ^18^, ^19^, ^20^, ^21^The planned establishment of the first CIRT center in the United States marks an important milestone for the field, creating new opportunities for clinical investigation and future integration of CIRT into U.S. oncology practice.

Previous publications have established important foundations for CIRT in the United States by summarizing the clinical evidence, estimating potentially eligible patient populations, and discussing national needs and efforts to re‐establish heavy‐ion therapy.^22^, ^23^, ^24^, ^25^Building on these contributions, the present work is organized as a two‐part review series centered on the Mayo Clinic Florida (MCF) program. The series addresses a complementary translational question: how should accumulated international clinical experience and the anticipated characteristics of the U.S. CIRT patient population inform the clinical requirements, biological dose framework, and technical design and implementation of the first U.S. CIRT program? Part I focuses on the clinical foundation for CIRT. We review the physical and radiobiological rationale for CIRT, summarize the current international clinical experience, discuss established and emerging disease indications, examine current approaches to biological dose modeling, and ultimately evaluate the clinical need and projected demand for CIRT in the United States. We also describe the biological dose calculation framework adopted by the MCF program to reproduce and compare established international CIRT dose prescription systems and support the application of accumulated international clinical experience in the first U.S. CIRT program. The companion part II article focuses on the implementation of the first U.S. CIRT center, describing the design philosophy and integrated clinical platform envisioned to meet the anticipated clinical demands of the U.S. CIRT patient population through the safe, accurate, and efficient clinical delivery of CIRT.

## 2 PHYSICS AND BIOLOGICAL RATIONALS FOR CIRT

The physical advantages of CIRT arise from the unique interactions of charged particles with matter. Figure 1 compares the intrinsic physical characteristics of proton and carbon ion beams with equivalent range in water. To illustrate the underlying particle interactions independent of beamline effects, the depth dose curve, linear energy transfer (LET), and lateral beam profiles were generated using MC simulations in water assuming monoenergetic beams with no energy spread and infinitesimal pencil beam spot size.

**FIGURE 1:**
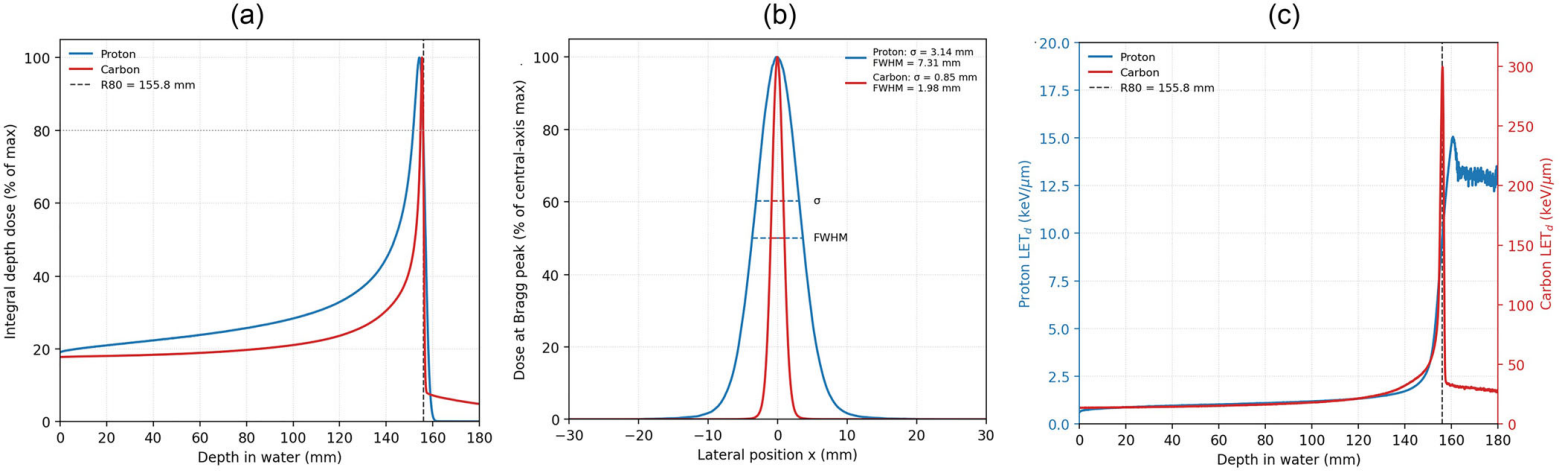
Comparison of the physical characteristics of proton and carbon ion beams with equivalent range in water. (a) Integrated depth‐dose (IDD) distributions for a 149 MeV proton beam and a 281.6 MeV/u carbon ion beam, illustrating the Bragg peak and the characteristic fragmentation tail of carbon ions. (b) Lateral dose profiles at the Bragg peak, demonstrating the reduced lateral penumbra of carbon ions resulting from decreased multiple Coulomb scattering. (c) linear energy transfer (LET) as a function of depth, showing the substantially higher LET of carbon ions, particularly near the distal region of the Bragg peak. All curves were generated using MC simulations in water assuming monoenergetic beams with no energy spread and infinitesimal pencil‐beam spot size to illustrate the intrinsic physical properties of the two particle species.

Like protons, carbon ions lose energy primarily through ionization and excitation as they traverse tissue, depositing a relatively low entrance dose followed by a sharp increase in energy deposition near the end of their range, known as the Bragg peak. This finite beam range and sharp distal dose falloff enable highly conformal dose delivery while minimizing irradiation of normal tissues beyond the target. Carbon ions, however, possess several physical characteristics that further enhance dose conformity compared with protons. Owing to their greater mass, carbon ions undergo substantially less multiple Coulomb scattering as they pass through tissue, resulting in a narrower lateral penumbra and improved sparing of adjacent organs at risk (OAR).

While both protons and carbon ions undergo nuclear interactions within tissue, fragmentation is substantially more pronounced for carbon ions because of their larger nuclear interaction cross section. These interactions generate lighter secondary fragments with longer ranges than the primary ions, producing a characteristic low‐dose fragmentation tail beyond the Bragg peak. Although the physical dose deposited by the fragmentation tail is substantially lower than the exit dose associated with photon beams, it represents an important consideration in treatment planning and biological dose calculation because the secondary fragments possess distinct LET and relative biological effectiveness (RBE) characteristics.^26^

Beyond the favorable physics property, carbon ions exhibit substantially greater biological effectiveness than photons and protons, primarily attributable to their high‐LET.^27^, ^28^High‐LET radiation deposits energy densely along the particle track, producing a microscopic pattern of highly localized ionization that differs fundamentally from the relatively sparse ionization events generated by low‐LET radiation.^29^As carbon ions decelerate along their path, LET increases progressively, reaching values of 100–200 keV/µm near the distal region of the Bragg peak.

The dense ionization pattern produced by high‐LET radiation induces clustered DNA lesions and complex double‐strand breaks that are substantially more difficult to repair than the isolated DNA damage typically caused by low‐LET radiation.^30^, ^31^, ^32^In addition, high‐LET radiation relies less on oxygen‐mediated free radical formation, resulting in a lower oxygen enhancement ratio (OER) than low‐LET radiation.^33^, ^34^Consequently, carbon ions maintain greater biological effectiveness in hypoxic tumor,^35^which are often resistant to conventional photon radiotherapy and are associated with poor clinical outcomes. High‐LET irradiation has also been shown to be effective against cancer stem cell populations and other intrinsically radioresistant tumor subpopulations.^36^, ^37^These physical and biological advantages provide the scientific foundation for CIRT and have driven its growing international clinical adoption.

## 3 GLOBAL CLINICAL EXPERIENCE AND CURRENT PRACTICE

### 3.1 Clinical development of CIRT

The clinical application of CIRT originated from pioneering heavy‐ion research at the Lawrence Berkeley National Laboratory, where helium, neon, and carbon ions were investigated for cancer treatment beginning in the 1970s. These early studies established the scientific feasibility of heavy‐ion radiotherapy and demonstrated its unique physical and biological advantages.^25^, ^38^However, following the closure of the Berkeley program in 1993, clinical development of heavy‐ion therapy in the United States came to an end, and subsequent leadership shifted to Japan and Europe.

A major milestone was the commissioning of the Heavy Ion Medical Accelerator in Chiba (HIMAC) at the National Institute of Radiological Sciences (NIRS; now the National Institutes for Quantum Science and Technology [QST]) in Japan in 1994, which established the first hospital‐based CIRT program dedicated to routine patient treatment.^14^Shortly thereafter, the Gesellschaft für Schwerionenforschung (GSI) in Darmstadt, Germany initiated clinical CIRT in 1997, establishing the foundation for the subsequent European CIRT program.^39^Over the following three decades, additional centers were established across Japan, Germany, Italy, China, Austria, and Korea, transforming CIRT from an experimental modality available at only a few research institutions into an established clinical treatment platform. As of 2026, seventeen CIRT centers are in clinical operation worldwide, and by the end of 2023, more than 57,000 patients had been treated with CIRT (https://ptcog.online; Accessed on July 17, 2026)

The global expansion of CIRT has been accompanied by continuous advances in accelerator and beam delivery technologies. Since the commissioning of HIMAC in 1994, accelerator technology has evolved substantially. The original HIMAC was a large‐scale dual synchrotron ring facility requiring extensive infrastructure and substantial capital investment.^40^Currently, all clinically operational CIRT facilities utilize synchrotron‐based accelerator systems. Carbon ions were initially delivered using single energy extraction (SEE) technique, in which only one energy is extracted and irradiated per spill. Subsequent developments introduced multiple‐energy extraction (MEE) allowing rapid energy switching within a single spill,^41^, ^42^thereby improving beam delivery efficiency. Accelerator development has also focused on progressively more compact and cost‐effective designs, including compact synchrotrons and superconducting magnet technology, which substantially reduced facility size while improving operational efficiency and treatment flexibility.^40^, ^43^More recently, accelerator development has expanded toward multi‐ion platforms capable of delivering carbon, helium, oxygen, and other ion species, providing the technological foundation for biologically optimized and personalized particle therapy.^44^, ^45^Although all clinically operational CIRT facilities currently use synchrotron‐based accelerators, the C400 compact superconducting cyclotron is under development in France as an alternative accelerator platform for CIRT.^43^, ^46^

In parallel with advances in accelerator technology, beam delivery techniques have also undergone substantial evolution. Early clinical carbon ion treatments were delivered using passive scattered beam, in which patient‐specific apertures were used to shape the lateral field and range compensators were used to conform the distal dose to the target.^40^A major technological advance occurred with the introduction of pencil‐beam scanning (PBS) at GSI in 1997, which fundamentally changed beam delivery by replacing passive beam shaping with magnetic beam scanning, allowing narrow pencil beams to paint the target spot‐by‐spot throughout the treatment volume.^39^The PBS enabled intensity‐modulated particle therapy (IMPT), providing improved 3D dose conformity, better sparing of adjacent OAR, and greater flexibility for treating complex target geometries.^47^

### 3.2 Clinical indications

In parallel with technological advances, the clinical practice of CIRT has also evolved substantially. Early clinical programs focused primarily on a limited number of rare, unresectable, and radioresistant malignancies, with treatment protocols established through prospective dose‐escalation and feasibility studies to define safe dose‐fractionation schedules and demonstrate clinical efficacy.^48^As clinical experience accumulated, indications progressively expanded to include a broader range of disease sites, recurrent tumors, and organ‐preservation strategies, establishing CIRT as a treatment option across multiple disease sites.^23^, ^49^, ^50^This expansion of clinical application was accompanied by increasingly rigorous clinical investigation, including prospective phase I/II studies and multicenter observational studies.^51^, ^52^, ^53^, ^54^Collaborative research networks, such as the Japan Carbon‐Ion Radiation Oncology Study Group (J‐CROS), together with multicenter prospective studies and standardized treatment protocols, enabled harmonization of clinical practice and the generation of higher‐level clinical evidence across institutions.^14^, ^16^

Based on the accumulated clinical experience in Asia and Europe, current indications for CIRT have largely been established for tumors characterized by intrinsic radioresistance, close proximity to critical OAR, locally advanced disease requiring safe dose escalation, or recurrent tumors requiring re‐irradiation.^13^, ^14^, ^15^, ^16^These indications reflect the physical and biological advantages of CIRT, including superior dose conformity and enhanced biological effectiveness. Representative disease sites include skull‐base chordoma and chondrosarcoma, adenoid cystic carcinoma and other non‐squamous head and neck malignancies, soft tissue and bone sarcomas, hepatocellular carcinoma, locally advanced pancreatic cancer, and locally recurrent rectal cancer, with additional experience reported for mucosal melanoma, selected non‐small cell lung cancer, gynecologic malignancies, and other challenging clinical cases.^23^, ^49^, ^50^, ^55^, ^56^

Across the established disease sites, CIRT has consistently demonstrated favorable local control with acceptable toxicity, particularly for tumors in which local treatment failure remains a major cause of morbidity and mortality despite advances in surgery, systemic therapy, and conventional radiotherapy.^51^, ^52^, ^53^, ^54^, ^55^, ^56^, ^57^While randomized clinical evidence remains limited, several ongoing prospective comparative trials are expected to further define the magnitude of clinical benefit for selected indications.^43^

### 3.3 Clinical RBE models and dose prescription systems

Because the biological effectiveness of charged particles differs from that of photon radiotherapy, treatment planning systems (TPSs) for particle therapy must account for RBE when converting physical dose into a clinically prescribed RBE‐weighted dose. In CIRT, LET changes dramatically along the carbon ion beam path, increasing from approximately 10 keV/µm in the entrance region to approximately 100–200 keV/µm near the distal Bragg peak. Because RBE depends strongly on LET,^27^the RBE varies substantially throughout the treatment field. Consequently, dedicated biophysical models are required to estimate spatial variations in RBE and enable biologically optimized treatment planning in CIRT.^28^

RBE modeling is therefore a defining feature of CIRT and forms the foundation of dose prescription, treatment planning, and the interpretation of clinical outcomes. Because the RBE‐weighted dose is derived from an underlying biophysical model, the reported clinical dose in Gy(RBE) is inherently model‐dependent. Accordingly, understanding the key concepts, clinical implementation, and clinical implications of RBE models is fundamental to interpreting the international clinical experience with CIRT. The evolution of RBE models has paralleled the development of clinical CIRT, from early passive scattering techniques to modern PBS and the emergence of distinct Japanese and European dose prescription systems. The clinical CIRT RBE models are summarized in Table 1.

**TABLE 1:** Summary of current clinical CIRT RBE models.

Model	Key concept	Clinical context	Reference data	Reference radiation	Clinical calibration	Typical clinical use
Mixed Beam Model (MBM)	Predicts biological effectiveness from the weighted contributions of monoenergetic beam components within the SOBP	Developed at NIRS for passive‐scattering carbon‐ion therapy	HSG cell survival experiments	Photons	Linked to historical fast‐neutron clinical experience through a clinical scaling factor	Historical passive‐scattering treatments
Modified Microdosimetric Kinetic Model (mMKM)	Predicts biological effectiveness from stochastic microscopic energy deposition	Developed at NIRS for PBS	HSG cell survival data experiment and microdosimetric simulation	Carbon‐ion reference beam	Biological dose normalized to preserve the historical NIRS clinical dose system	Clinical standard in Japan and most Asian facilities
Local Effect Model (LEM I)	Predicts biological effectiveness from local dose deposition and photon response survival curves	Developed at GSI for PBS	Photon cell‐survival curve and ion track structure	Photons	Direct calculation of photon‐referenced RBE‐weighted dose	Clinical standard in Europe

The mixed beam model (MBM) was the first RBE model implemented in routine clinical practice at NIRS for treatment planning and clinical application of passive scattering CIRT. Because a spread‐out Bragg peak (SOBP) consists of a mixture of primary and secondary particles with different energies and LET values, the MBM estimates the biological effect of the mixed radiation field from the LET‐dependent responses of its constituent monoenergetic beam components.^58^, ^59^Effective linear‐quadratic (LQ) parameters are related to LETd (computed including the contributions of all ions) and used to calculate the biological effectiveness across the SOBP. For clinical implementation, the MBM employed a two‐step dose prescription system. First, the model was calibrated using human salivary gland (HSG) cells and an endpoint of 10% cell survival to generate RBE depth profiles within the SOBP. HSG cells were selected because of their intermediate radiosensitivity across a panel of cell lines for representative of normal tissues and tumors. Second, the HSG cell‐based RBE profile was rescaled to clinical RBE using a clinical scaling factor of 1.44,^60^thereby anchoring the model to NIRS's prior clinical experience with fast‐neutron radiotherapy.

Following the introduction of PBS, NIRS redefined the biological modeling component of its clinical dose system using the modified microdosimetric kinetic model (mMKM). This transition enabled biologically optimized treatment planning for scanned carbon ion beams while preserving continuity with the clinical experience accumulated under the MBM.^61^, ^62^, ^63^The mMKM is based on the microdosimetric kinetic theory, which relates radiation‐induced cell killing to stochastic microscopic energy deposition within subcellular domains.^64^, ^65^, ^66^The model predicts increases in the linear (α) component of the LQ model with increasing LET through the saturation‐corrected dose‐mean specific energy (z*_D_), accounting for the overkill effect observed at very high LET.^63^To maintain consistency with historical clinical experience, the biological dose calculated by the mMKM is normalized to a reference carbon‐ion beam and converted to the established NIRS clinical dose scale using a fixed clinical scaling factor of 2.41.^61^Thus, the transition from MBM to mMKM changed the underlying biological modeling framework without changing the established NIRS clinical dose scale. Following its clinical introduction at NIRS, the mMKM‐based system was subsequently adopted by several CIRT centers across Asia. Although several extensions have been proposed to incorporate additional biological mechanisms such as hypoxia, DNA damage complexity, and dose‐rate effects and cell cycle redistribution,^67^, ^68^, ^69^, ^70^, ^71^these developments have primarily served as research models, whereas the original mMKM remains the model used for routine clinical CIRT in Japan and many other Asian centers.

In parallel, investigators at GSI independently developed the Local Effect Model (LEM) as the biological basis for PBS CIRT in Europe. LEM is a track‐structure‐based radiobiological model, and the key assumption of the model is that equal local energy depositions produce the same local biological effect, irrespective of whether the energy is deposited by photons or carbon ions. Accordingly, the enhanced biological effectiveness of high‐LET radiation is attributed to the highly heterogeneous microscopic dose distribution surrounding ion tracks.^72^, ^73^, ^74^The LEM predicts biological effectiveness by combining photon‐derived cell survival characteristics with the microscopic spatial dose distribution produced by ion tracks. The resulting LEM I framework was translated into clinical practice through its implementation in a TPS, where it became the radiobiological basis for carbon‐ion treatment planning in Europe.^75^This implementation established a photon‐referenced RBE‐weighted dose prescription system that differs fundamentally from the Japanese approach. Several subsequent refinements (LEM II–IV) have been introduced to improve the description of DNA damage and track structure,^76^, ^77^, ^78^, ^79^however, LEM I remains the version used for routine clinical CIRT treatment planning in European CIRT centers.

### 3.4 Clinical implications of different RBE models

The clinical implementation of CIRT has evolved along two principal regional pathways. The Japanese dose prescription system was initially established using the MBM and later transitioned to the mMKM while preserving the established NIRS clinical dose scale. European centers adopted the independently developed, photon‐referenced LEM system. Today, most centers in Asia use mMKM‐based dose prescription system, whereas LEM I remains the predominant clinical model in Europe.

Because these systems differ not only in their underlying radiobiological models but also in their model parameters, reference radiation, calibration and scaling conventions, and clinical implementation, they may predict different RBE values and spatial biological dose distributions under the same irradiation conditions.^80^, ^81^, ^82^, ^83^, ^84^Consequently, although both systems report dose in Gy(RBE), the nominal dose values are not directly interchangeable. The absence of a common dose scale complicates comparisons of clinical outcomes, limits the direct transfer of treatment protocols between institutions, and presents a major challenge for multinational clinical trials and pooled analyses.^85^To address these issues, considerable effort has been devoted to developing translation strategies between dose prescription systems, including model‐ and indication‐specific prescription conversion factors, MC‐based recalculation methods, and translated OAR constraints that facilitate biologically equivalent treatments across institutions.^80^, ^86^, ^87^However, such conversions are not universally applicable and depend on prescription dose, treatment site, target geometry and depth, beam configuration, and the biological endpoint.^80^, ^85^, ^86^

## 4 INTEGRATION OF INTERNATIONAL CLINICAL EXPERIENCE INTO THE FIRST U.S. CIRT PROGRAM

To support the development of the first clinical CIRT facility in the United States, the MCF program adopted a biological dose calculation framework to facilitate the application of established international clinical experience with CIRT. The first step was to reproduce the two clinical RBE models underlying the current Asian and European CIRT dose prescription systems: the mMKM and the LEM I. Both models were independently implemented within the MCF computational framework to enable direct in silico comparison of biological dose calculations and treatment plans. In parallel, the Mayo Clinic Florida Microdosimetric Kinetic Model (MCF MKM)^88^, ^89^was developed to support biological dose calculation within the MCF computational framework while maintaining compatibility with the clinically established mMKM dose prescription system. In a recent study, treatment plans generated using the MCF MKM showed good agreement with those generated using the clinical mMKM implemented at QST, supporting the ability of the MCF framework to reproduce the Japanese clinical dose prescription system.^90^

A second objective was to determine whether the same clinically established RBE model and dose prescription system would produce equivalent biological dose distributions when implemented using different institution‐specific treatment delivery systems. This question is particularly important because differences in beam delivery nozzle design may alter the microdosimetry quality used for biological dose calculation, while the detailed nozzle designs are generally proprietary and not publicly available. To address this question, two collaborative studies were conducted with MedAustron and the Heidelberg Ion‐Beam Therapy Center (HIT). In each study, the respective beam delivery system was modeled in MC, and the resulting biological dose distribution was compared with that predicted for the MCF beam delivery system. Both studies showed good agreement in the predicted biological dose distributions despite substantial differences in nozzle design, indicating that nozzle‐specific differences have only a minor impact on biological dose prediction. The MedAustron study has been published,^91^while the corresponding HIT manuscript is currently in preparation. These studies demonstrate that established dose prescription systems can be reproduced with close agreement within the MCF framework. This capability provides a foundation for MCF treatment planning, enabling the integration of extensive clinical experience and accumulated evidence from CIRT centers worldwide into a unified and clinically informed treatment‐planning framework.

Additionally, this framework provides a basis for continued collaboration with established CIRT centers. Such collaboration may facilitate cross institutional treatment plan comparison, dosimetric and radiobiological benchmarking, translation of clinical protocols and the development of multicenter clinical studies. As clinical CIRT is established in the United States, such collaborations will be important not only for leveraging the extensive experience accumulated internationally, but also for contributing U.S. clinical and technical experience to ongoing efforts toward harmonization and evidence generation across the global CIRT community.

## 5 CIRT IN THE UNITED STATES

### 5.1 Demand for CIRT

Despite more than three decades of clinical experience with CIRT in Asia and Europe, patients in the U.S. currently have no domestic access to this treatment modality. As a result, U.S. patients who may benefit from CIRT must either pursue treatment overseas or forego the treatment altogether. This limitation has also prevented meaningful participation of U.S. patients in clinical trials evaluating the effectiveness of CIRT.

Several studies have evaluated the potential demand for CIRT within the U.S. Using national cancer database incidence data together with disease‐specific eligibility criteria derived from established international CIRT protocols, Malouff et al.^24^ estimated the number of U.S. patients potentially eligible for CIRT. Adaptation rates derived from existing carbon ion treatment programs were subsequently applied to project the number of patients likely to receive CIRT in clinical practice, resulting in approximately 89,946 patients being projected as eligible for CIRT in 2019, with this number expected to increase to 114,823 patients annually by 2025. Complementing this analysis, Foote et al. used a conservative estimate based on surveillance, epidemiology, and end results (SEER) cancer statistics and reported that approximately 44,340 patients annually in the United States have malignancies that may benefit from CIRT, yet only 15 U.S. citizens had traveled abroad to receive CIRT between 1994 and 2020.^22^

International studies published in recent years provide additional context, although estimates vary according to the indications considered, assumptions regarding clinical adoption, and health‐system context. In Australia, Ahern et al. estimated that nearly 1400 patients annually, approximately 1% of all cancer patients or 2% of patients recommended for radiotherapy, may preferentially benefit from CIRT.^92^In Korea, Cho et al. estimated a realistic annual CIRT need of approximately 4000–6300 patients, corresponding to 2–3% of newly diagnosed cancer patients.^93^A recent UK critical review also identified several tumor sites for which national cancer incidence and mortality patterns support a clinical need for CIRT, although it did not provide a single population‐wide eligibility estimate.^50^

### 5.2 Anticipated CIRT patient population

Beyond the overall projected demand for CIRT in the United States, understanding the anticipated characteristics of the future CIRT patient population is equally important because these patients will ultimately define the clinical and technical requirements of the first U.S. CIRT center. Using disease‐specific eligibility criteria derived from established international CIRT protocols together with adaptation rates from existing carbon ion programs, Malouff et al. projected the disease distribution of the anticipated U.S. CIRT patient population in 2025.^24^

After excluding prostate cancer because of the uncertain future role of CIRT in this disease, the projected U.S. CIRT patient population is dominated by non‐small cell lung cancer, followed by hepatocellular carcinoma, soft tissue sarcomas, cholangiocarcinoma, pancreatic cancer, glioblastoma, and selected head and neck malignancies (Figure 2), representing an anticipated annual patient population of approximately 34,000 patients. These disease sites share several clinically important characteristics, including respiratory motion, anatomical variation during treatment, close proximity to critical OAR, and frequent need for adaptive treatment. which place exceptional demands on treatment delivery and treatment management.

**FIGURE 2:**
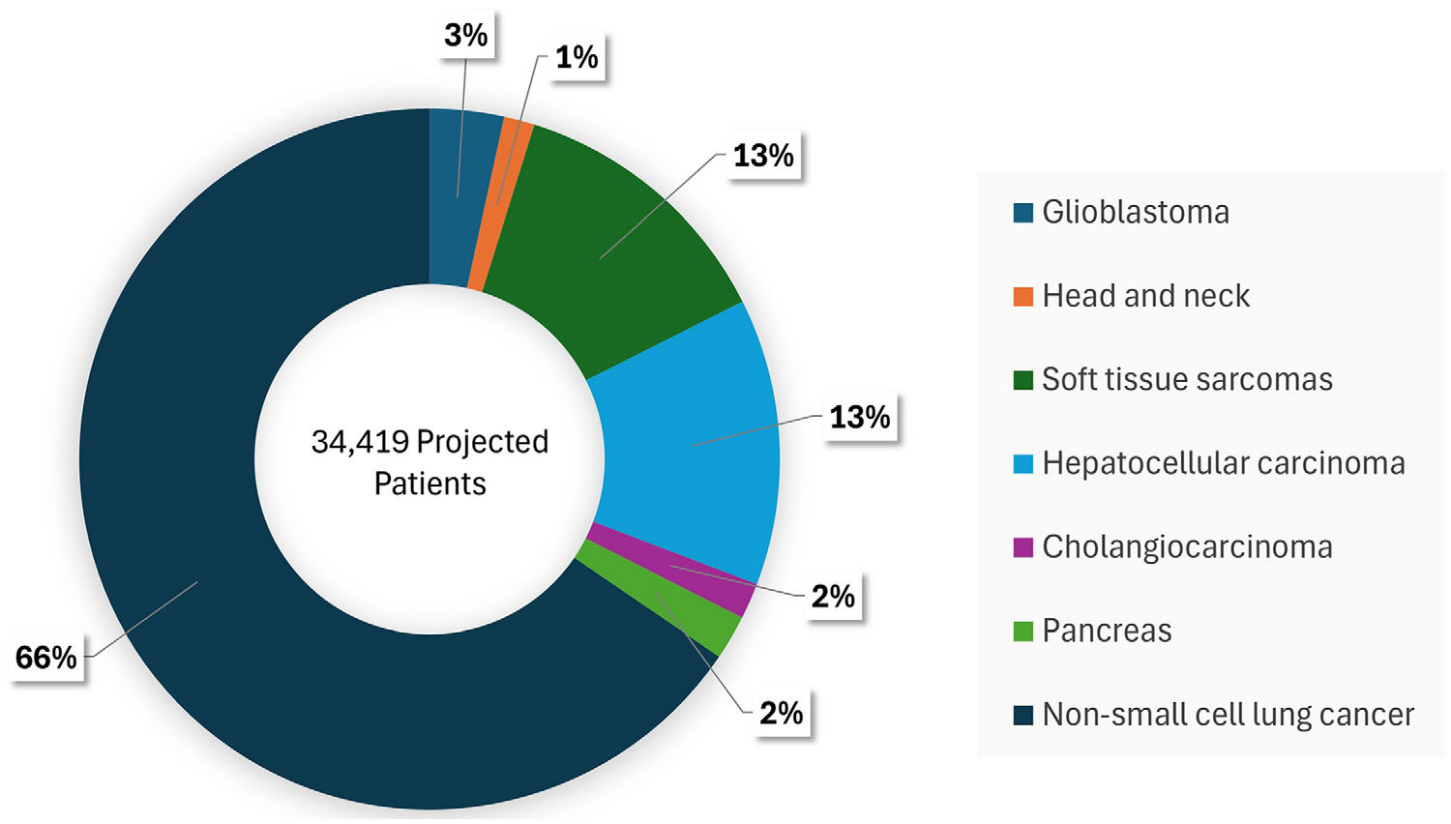
Projected distribution of the anticipated U.S. CIRT patient population in 2025 based on disease‐specific adaptation‐corrected estimates reported by Malouff et al.,^24^ excluding prostate cancer. Percentages represent the proportion of the projected 34,419 patients eligible for CIRT after excluding prostate cancer. Adapted from Table 4 of Malouff et al.^24^

### 5.3 The First U.S. CIRT facility

Recognizing both the substantial unmet clinical need for CIRT and the growing national effort to re‐establish heavy‐ion therapy in the United States, Mayo Clinic Florida (MCF) is developing the first clinical CIRT facility in North America. The project builds upon more than a decade of institutional commitment to particle therapy and carbon ion research, including early internal evaluations of CIRT, international collaborations with the NIRS/QST. It is being developed as part of an integrated oncology initiative designed to support clinical care, translational research, and prospective clinical trials. Construction of the facility was announced in 2019 and groundbreaking occurred in 2022, marking a major milestone in the reintroduction of clinical CIRT to the United States. Clinical proton therapy is projected to commence in 2027, followed by carbon ion therapy in 2028.

Figure 3 illustrates the layout of the MCF particle therapy facility. The accelerator system consists of dedicated proton and carbon linear accelerators, which accelerate proton beams to 7 MeV and carbon ions to 4 MeV/u, respectively, for injection into the hybrid synchrotron. The synchrotron operates with either proton or carbon beams, accelerating the selected particle species to clinical treatment energies of 70–230 MeV for protons or 100–430 MeV/u for carbon ions. The accelerator system is designed to accommodate additional ion species through the addition of dedicated ion sources and corresponding control system modifications. The extracted beams are transported through the high‐energy beam transport system to three treatment rooms in the initial clinical configuration, including two proton rotating gantry rooms and one fixed‐beam treatment room capable of delivering both proton and carbon ion therapy. The two proton gantry rooms are equipped with integrated image‐guidance systems, including kV planar radiographic imaging and kV cone‐beam CT (CBCT), with support for 2D/2D, 2D/3D, and 3D/3D image registration. In addition, fluoroscopic imaging is available at user‐selectable frame rates to enable real‐time image‐based gating. The fixed‐beam room is equipped with a CT‐on‐rails system, a pair of orthogonal X‐ray imaging systems, and a pair of oblique X‐ray imaging systems, supporting 2D/2D, 2D/3D, and 3D/3D image registration, fluoroscopic imaging, and real‐time gating. In addition, the facility is designed with future expansion capability to incorporate two carbon ion rotating gantry treatment rooms.

**FIGURE 3:**
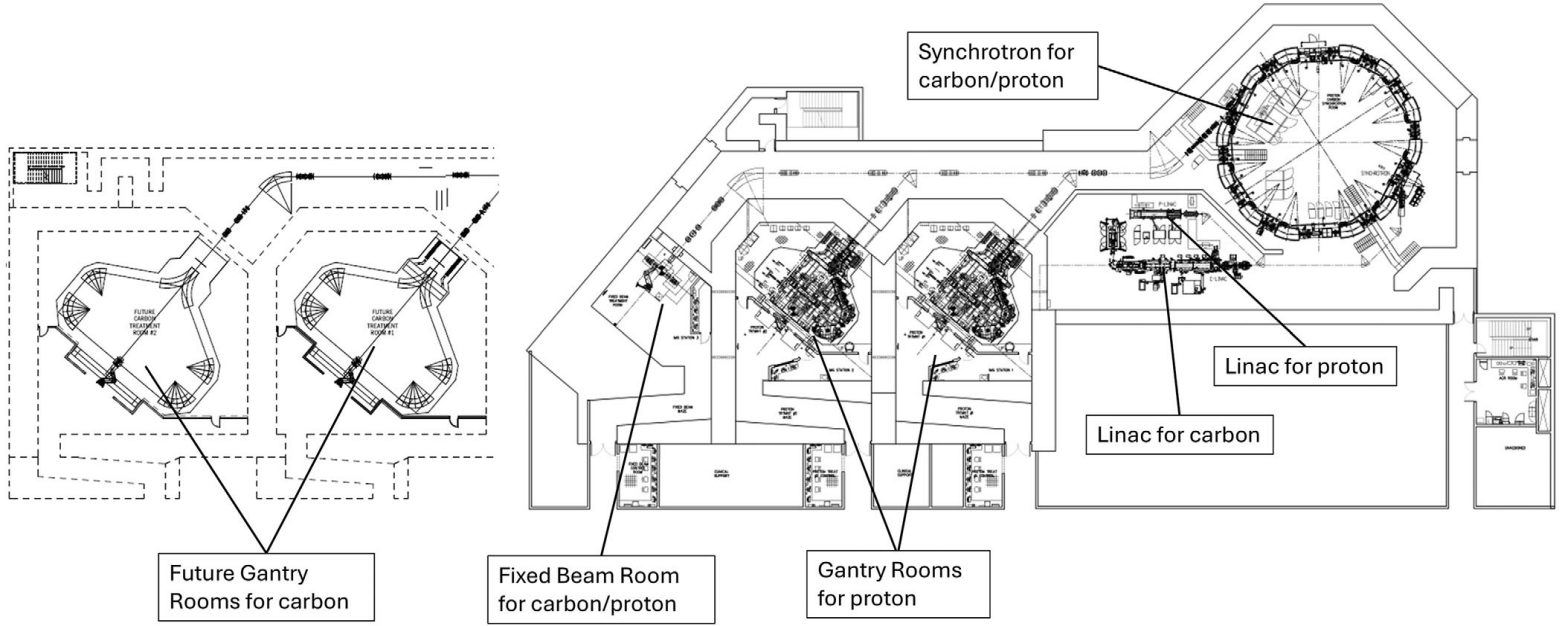
Schematic overview of the Mayo Clinic Florida (MCF) particle therapy facility, illustrating the hybrid synchrotron system, treatment rooms, and planned future expansion for carbon ion gantry rooms.

Importantly, the Mayo Clinic approach extends beyond establishing domestic access to CIRT. Development of the first U.S. carbon ion facility provides an opportunity to design the MCF program around the anticipated clinical needs of its future patient population. The anticipated patient population encompassing many clinically complex disease sites requiring accurate, highly conformal, and adaptive treatment, places great demands on treatment management and the supporting technologies required to deliver CIRT safely, accurately, and efficiently. These anticipated clinical demands provide the foundation for the design philosophy of the first U.S. CIRT center, which is described in the companion article.

## 6 CONCLUSION

Over the past three decades, CIRT has evolved from an experimental treatment available at a few research centers into an established clinical modality supported by substantial clinical experience. Continuous advances in accelerator technology, beam delivery, treatment planning, image guidance, and radiobiological modeling, together with growing clinical evidence, have enabled CIRT to become an important treatment option for selected malignancies in which conventional radiotherapy remains limited by either physical dose constraints or insufficient biological effectiveness.

The accumulated evidence demonstrates that the clinical role of CIRT is now well established for a range of challenging disease sites, particularly those characterized by intrinsic radioresistance, proximity to critical organs, locally advanced disease requiring safe dose escalation, and recurrent tumors requiring re‐irradiation. Although important challenges remain, including continued refinement of RBE modeling, standardization of treatment protocols, and generation of higher‐level clinical evidence through prospective comparative trials, the field has matured substantially over the past three decades and continues to expand through international collaboration and technological innovation.

The anticipated demand for CIRT in the U.S. provides a compelling rationale for establishing domestic CIRT capability. Importantly, the anticipated U.S. CIRT patient population defines the clinical demands that should shape the design of the first U.S. CIRT center. Rather than simply replicating existing CIRT centers, the first U.S. CIRT center provides an opportunity to develop an integrated clinical platform tailored to these anticipated future clinical demands. The resulting design philosophy and its implementation in the MCF CIRT program are presented in the companion article.

## AUTHOR CONTRIBUTIONS

Conceptualization, Xiaoying Liang and Chris J Beltran; Writing‐original draft, Xiaoying Liang; Writing‐review & editing, Chris J Beltran and Xiaoying Liang; Both authors have read and approved the final manuscript.

## CONFLICT OF INTEREST STATEMENT

The authors declare no conflicts of interest.

## Data Availability

The data that supports the findings of this study are available from the corresponding author upon reasonable request
